# Preparation and evaluation of transdermal permeation of Huperzine A ethosomes gel in vitro

**DOI:** 10.1186/s40360-024-00742-w

**Published:** 2024-02-26

**Authors:** Jiyu Wu, Renai Xu, Xiaowei Xu, Shiyuan Ye, Aifang Huang

**Affiliations:** Department of Pharmacy, The First Affiliated Hospital of Wenzhou Medical University, Wenzhou, Zhejiang 325000 People’s Republic of China

**Keywords:** Huperzine A, Ethosomes, Gel, Transdermal permeation

## Abstract

This study aimed to design and evaluate the transdermal permeation of Huperzine A ethosomes gel in vitro. Huperzine A ethosomes were prepared using the injection method, and their physical and chemical properties were characterized. A comparison was made between Huperzine A ethosomes gel, ordinary gel, and cream. The Franz diffusion cell test on mouse abdominal skin was conducted, and Huperzine A concentration was determined using LC-MS/MS. Transdermal volume, skin retention, and transdermal rate were used to assess the percutaneous permeability of the three preparations. Results demonstrated that Huperzine A ethosomes gel exhibited significantly higher accumulative permeation, transdermal rate, and skin retention compared to ordinary gel and cream. The findings suggest that Huperzine A ethosomes gel, with its controllable quality and favorable transdermal absorption properties, holds potential as a safe option for clinical administration.

## Introduction

Alzheimer’s disease (AD) is a progressive and irreversible condition characterized by memory, cognition, and executive function decline. As the global aging population increases, AD has emerged as a prominent neurodegenerative disease [1]. Despite its multifactorial nature, the etiology and pathogenesis of AD remain elusive, and there are no universally effective treatments or cures. Traditional Chinese medicines have shown promise in preventing and ameliorating AD. Huperzine A, an alkaloid derived from *Huperzia serrata*, originally used for myasthenia gravis treatment, has demonstrated memory-enhancing effects in elderly individuals with mild memory impairment. It acts as a potent, selective, and reversible central acetylcholinesterase inhibitor [2]. Preclinical studies have reported Huperzine A’s ability to reverse cognitive impairment in animal models and improve learning and memory deficits in humans [3, 4].

The clinical safety of Huperzine A, an active component extracted from traditional Chinese medicine, is well-established. However, at high doses, adverse reactions, particularly in the digestive system, still occur, which is a major limitation for its use [5]. Furthermore, Huperzine A is rapidly metabolized and has a short residence time in the body after oral or injection administration [2]. To maintain therapeutic efficacy, frequent dosing is required. Developing a transdermal formulation can effectively address the challenges of sustained drug delivery and reduce adverse reactions in the digestive system. Ethosomes, as carriers for transdermal delivery of active components in traditional Chinese medicine, have gained considerable attention in formulation research in recent years. Ethosomes can improve the solubility, stability, and absorption of active components, thereby enhancing therapeutic effects [6–8]. Formulating Huperzine A into an ethosomes gel can reduce skin irritation, increase stability, and enhance transdermal drug absorption.

In light of widespread memory issues among patients, strict adherence to multi-dose regimens becomes challenging, leading to significant fluctuations in blood drug concentrations and potential adverse reactions. The development of a transdermal formulation for Huperzine A aims to maintain effective blood drug levels, ensure sustained therapeutic effects, and mitigate the peak-trough concentration fluctuations associated with multiple oral administrations. This not only enhances clinical significance but also improves patient compliance. Here, we developed Huperzine A-loaded solid lipid nanoparticles in gel form and evaluated their in vitro transdermal permeation characteristics. This study establishes an experimental basis for future investigations on transdermal delivery of Huperzine A.

## Instruments and materials

### Instruments

Ultra performance liquid chromatography tandem mass spectrometry (ACQUITY I-S) was obtained from waters Co (Waters, US). Thermo biofuge primo Bench Centrifuge was obtained from Thermo Fisher Scientific (China) Co (Thermo Fisher, China). Collector type constant temperature heating magnetic stirrer (DF-101S 2L) was purchased from Lingke Industrial Development Co., Ltd (Shanghai, China). Electronic balance (AB135-S/FACT) was obtained from Mettler Toledo instruments Co., Ltd (METLER TOEDO, Switzerland). Franz diffusion cell (TT-18) was purchased from Zhengtong Technology Co., Ltd. (Tianjin, China). Desktop pH meter (FE28-standard) was obtained from Mettler Toledo instruments Co., Ltd (METLER TOEDO, Switzerland). Particle size analysis was performed using the Malvern Zetasizer Nano-S90 laser particle size analyzer (Malvern, UK). The transmission electron microscope (TEM) used in this study was the Talos L120C model from FEI Company (USA). SCIENTZ-IID Ultrasonic Cell Disruptor (Ningbo Scientz Biotechnology Co., Ltd., China) was acquired from Ningbo Scientz Biotechnology Co., Ltd (China).

### Drugs and reagents

Huperzine A (batch No. 21072206, purity 99.8%) was obtained from Shanghai Tongtian Biotechnology Co., Ltd., (Shanghai, China). Soybean lecithin (batch No. so-210803) was purchased from Shenyang Tianfeng biopharmaceutical Co., Ltd. (Liaoning, China). Carbomer 940 was from Tangyao Biotechnology Co., Ltd. (Shanxi, China). Potassium dihydrogen phosphate was from Guangdong Guanghua Technology Co., Ltd. (Guangdong, China). Disodium hydrogen phosphate dodecahydrate was from Xilong Science Co., Ltd. (Guangdong, China). All other ingredients used were of analytical grade.

### Animals

Thirty CL grade C57BL/6 male mice weighting 20 ± 2 g were obtained from Zhejiang Academy of Medical Sciences with an animal license No: SYXK (Zhejiang) 2019–0011.

## Methods and results

### Preparation of Huperzine A ethosomes

The ethanol injection method was used [9, 10] to prepare the ethosome formulation. Optimization experiments revealed that 30% ethanol content in the ethosome was optimal. Soybean phospholipids (3 g), Huperzine A (8 mg), and anhydrous ethanol (30 g) were accurately weighed and dissolved in a 100 ml graduated cylinder, resulting in the ethanol phase. Sixty-seven grams of PBS buffer solution (pH 7.4) were taken in a conical flask, serving as the aqueous phase. Under magnetic stirring (40 °C, 500 rpm), the ethanol phase was slowly injected into the aqueous phase. After complete addition of the ethanol phase, stirring (40 °C, 500 rpm) continued for an additional 30 min under sealed conditions (Sealed with a sealing film). The resulting alcohol liposomes were subjected to 60 s of ultrasonication (150 W, 50 Hz) in an ice bath, followed by filtration through a 0.22 μm membrane. The solution was stored in a light-shielded, sealed container at 4 °C.

### Characterization of Huperzine A ethosomes

#### Determination of particle size, polydispersity index, zeta potential, and encapsulation efficiency

An appropriate amount of the Huperzine A ethosomes prepared under section “Preparation of Huperzine A ethosomes” was taken and diluted to the desired concentration with physiological saline. The particle size, polydispersity index (PDI), and zeta potential were measured using a Malvern particle size analyzer.

Encapsulation efficiency was determined using chitosan column chromatography [11]. Three parallel samples (0.2 g each) of Huperzine A ethosomes prepared as described in section “Preparation of Huperzine A ethosomes” were applied to fully swollen and packed chitosan gel microcolumns. Each column was washed four times with 0.2 mL of purified water, and the collected eluents were combined. After adding 5 mL of methanol and sonication for 30 min, the solution was adjusted to 10 mL with methanol and filtered through a 0.22 μm filter membrane. The content was analyzed using the chromatographic conditions described in section “Chromatographic and mass spectrometric conditions” to calculate the mass of Huperzine A encapsulated in the ethosomes (C_encap). An additional sample of 0.2 g was taken, adjusted to 10 mL with methanol, sonicated for 30 min, and filtered through a 0.22 μm filter membrane. The content was analyzed using the chromatographic conditions described in section “Chromatographic and mass spectrometric conditions” to calculate the total mass of Huperzine A in the sample (C_total). The encapsulation efficiency of Huperzine A in the ethosomes was calculated as C_encap/C_total × 100%. The mean encapsulation efficiency of Huperzine A was determined to be 77.05% (75.23–78.55%).

The results showed that the Huperzine A ethosomes was a yellowish, semi-transparent liquid with a noticeable light blue opalescence. The prepared ethosomes had a small particle size with a narrow particle size distribution range. The polydispersity index was less than 0.3, indicating that the nanoparticle size distribution was relatively uniform. The zeta potential was around 25.6 mV, indicating good stability and resistance to aggregation. The encapsulation efficiency was approximately 77.05%. The results are presented in Table 1.

**Table 1:** Characterization of the Huperzine A loaded ethosomes (${\bar{\text{x}}} \pm {\text{s}}$, n = 3)

Particle size (nm)	Polydispersity	index Zeta potential (mv)	Encapsulated efficiency (%)
62.6 ± 1.7	0.242 ± 0.012	25.60 ± 0.20	77.1 ± 1.8

#### Morphological characterization of Huperzine A ethosomes

**Fig. 1:**
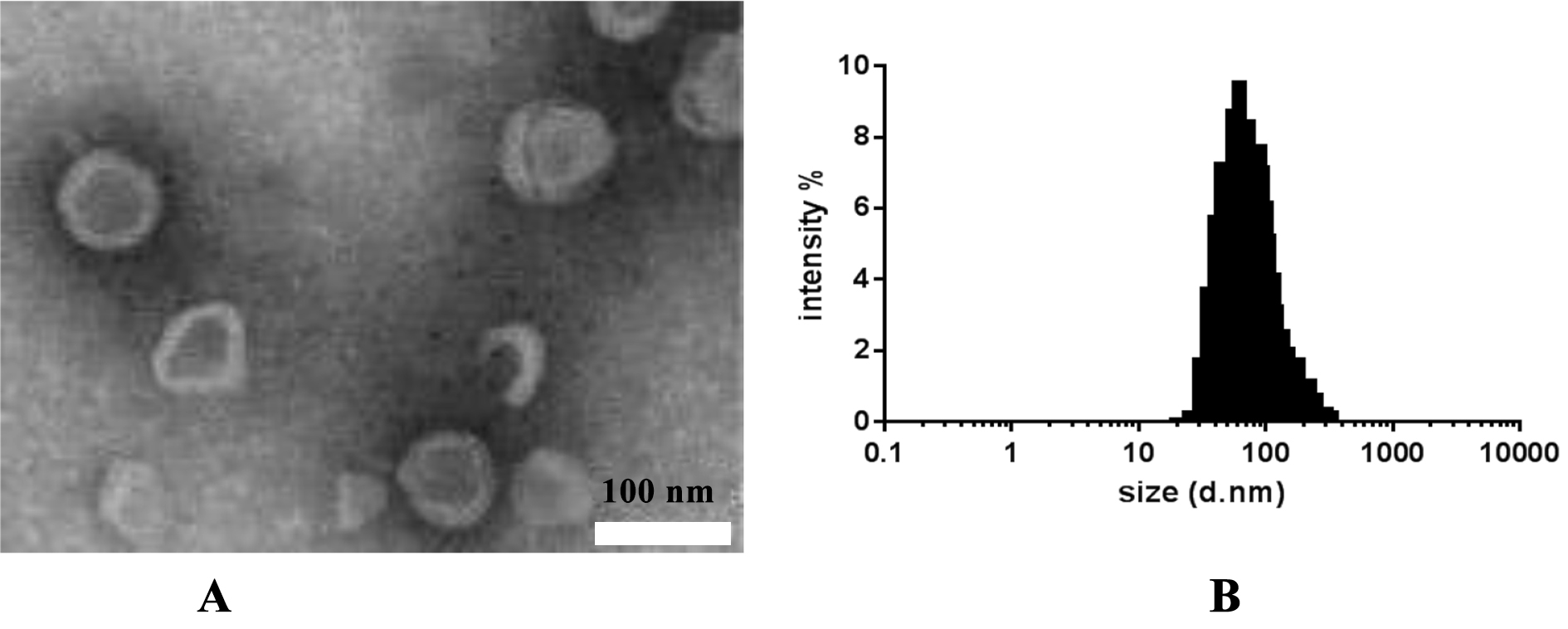
Characterization of ethosomes (**A**) TEM of ethosomes; (**B**) size distribution

The Huperzine A ethosomes prepared in section “Preparation of Huperzine A ethosomes” were diluted with pure water and drop-cast onto a copper grid (mesh size: 200), followed by air-drying. The sample was negatively stained with 2% uranyl acetate and air-dried. The resulting sample was observed under a transmission electron microscope, revealing nearly spherical particles with a vesicular structure, consistent with the literature on ethosome particles [12] (Fig. 1). The particle size was approximately 60 nm, and the size distribution was uniform, in agreement with the measurements obtained using a particle size analyzer.

### Preparation and property investigation of Huperzine A ethosomes gel

#### Preparation of ethosomes gel, ordinary gel, and cream

2 g of Carbomer 940 was mixed with 98 g of 30% ethanol aqueous solution and allowed to swell for 24 h. The pH was adjusted to 7.4 using triethanolamine to obtain a blank gel [13]. For the preparation of the ordinary gel, Huperzine A was dissolved in a 30% ethanol aqueous solution to create a solution containing 80 μg of Huperzine A per gram. This solution was then added to the blank gel in a 1:1 ratio and thoroughly stirred to obtain the ordinary gel.

To prepare the ethosomes gel, the method of ethanol injection described in section “Preparation of Huperzine A ethosomes” was used to prepare the ethosomes. The ethosomes was then added to the blank gel matrix in a 1:1 ratio and continuously stirred until a homogeneous gel was formed.

The oil phase, consisting of hard fatty acid, liquid paraffin, and solid paraffin, was mixed. The aqueous phase, containing Huperzine A dissolved in anhydrous ethanol, glycerol, triethanolamine, and purified water, was prepared. Both phases were heated to 75 °C and held for 20 min. The aqueous phase was slowly added to the oil phase under stirring at 5000 rpm for 10 min in a 75 °C water bath. High-speed stirring was continued until the emulsion cooled to room temperature, resulting in the formation of Huperzine A cream [14].

Each gram of the formulation contains 40 μg of Huperzine A.

#### **Investigation of basic properties of Huperzine A ethosomes gel** [15, 16]

The Huperzine A ethosomes gel exhibited uniformity and had a pale yellow, semi-transparent appearance. Dissolving approximately 1 g of the sample in a 30% ethanol solution and diluting it to 10 mL, the pH was measured to be approximately 6.4 using a pH meter. Centrifuging approximately 5 g of the sample in a graduated centrifuge tube at 3000 r · min^−1^ for 30 min did not result in any layering phenomenon. The heat resistance stability of three batches of the sample was tested by sealing them in centrifuge tubes and incubating them at 55 °C for 6 h, showing no observed layering phenomenon. Similarly, the cold resistance of another three batches of the sample was tested by sealing them in centrifuge tubes and storing them at −20 °C for 24 h, also without any observed layering phenomenon.

### Determination of Huperzine A

#### Chromatographic and mass spectrometric conditions [17, 18]

Chromatographic conditions: Acquity UPLC BEH C18 column (2.1 mm × 50 mm, 1.7 μm); mobile phase: A is acetonitrile, B is 0.1% formic acid water; gradient elution: 0–0.5 min, A:B (10:90); 0.5–1.0 min, A:B (90:10); 1.0–1.4 min, A:B (90:10); 1.4–1.5 min, A:B (10:90); 1.5–2.0 min, A:B (10:90); injection volume 4 μL; flow rate 0.4 mL · min^−1^; column temperature 40 °C.

The mass spectrometry conditions were as follows: Electrospray ionization source (ESI) and multiple reaction monitoring (MRM) mode were employed with positive ion detection. The source emission voltage was 5500 V, the ion source temperature was 450 °C, the curtain gas (CUR) pressure was 15 psi (1 psi = 6.895 kPa), the nebulizer gas (Gs1) pressure was 50 psi, and the auxiliary heater (Gs2) pressure was 55 psi. Additional parameters can be found in Table 2.

**Table 2:** Monitoring parameters of multi reaction on Huperzine A

Compound	Parent ion	Declustering potential (DP)/V collision energy (CE)/eV		Fragment ion
Huperzine A	242.79	10	20	226.1

#### Methodological investigation

Preparation of reference solution,test solution of Huperzine A ethosomes gel, and blank ethosomes gel solution were performed to investigate the method’s specificity, linearity, intra-day and inter-day precision, and recovery of samples.

**Fig. 2:**
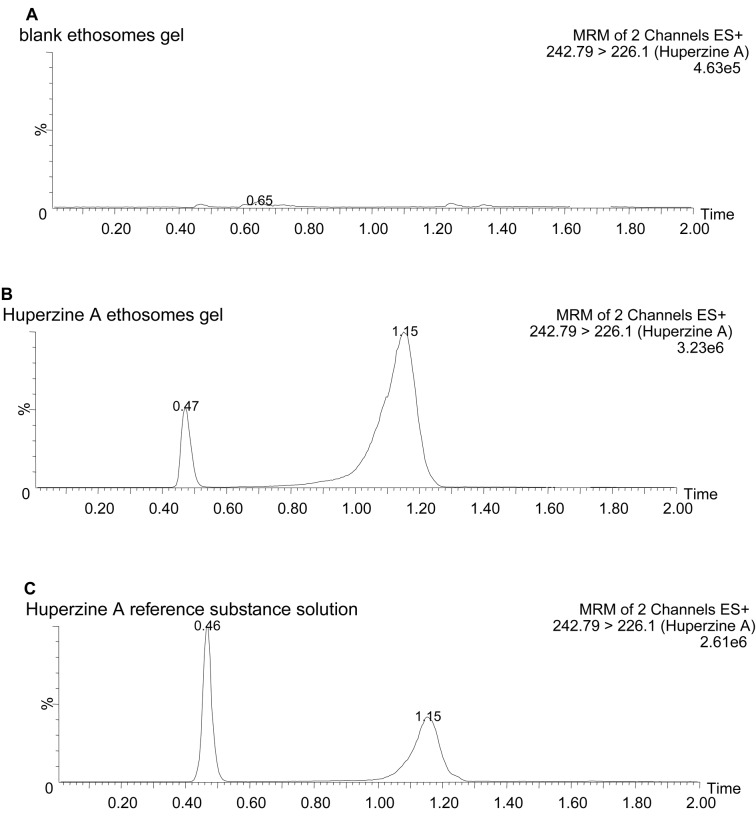
MRM chromatogram of Huperzine A

Figure 2 demonstrates the specificity results, confirming the excellent specificity of Huperzine A in the ethosomes gel test solution without interference from excipients. Huperzine A exhibited a linear relationship within the concentration range of 0.02–2 μg · ml^−1^, with the regression equation A = 795.53C + 2279.34 (*r* = 0.9970), indicating good linearity between the concentration (C) and the peak area (A).

In the precision study, the low, medium, and high quality reference solutions with concentrations of 0.06, 0.2, and 1.2 µg · mL^−1^, respectively, were analyzed in five replicates for intra-day and inter-day precision. The intra-day precision exhibited an RSD of ≤0.72%, while the inter-day precision showed an RSD of ≤1.82%. The mean recovery rates for the low, medium, and high concentrations were 104.0, 101.9, and 98.3%, respectively, with an RSD of 2.7%, indicating favorable precision and stability of the method.

### In vitro percutaneous permeation experiment

#### Percutaneous penetration test

For the study, we obtained thirty clean grade C57BL/6 male mice weighing 20 ± 2 g from Zhejiang Academy of Medical Sciences, with an animal license No: SYXK (Zhejiang) 2019-0011. As per ethical guidelines, we ensured humane euthanasia of the mice. To achieve this, we administered pentobarbital sodium as the anaesthetic agent via intraperitoneal injection at a dosage of 150–200 mg/kg. The use of pentobarbital sodium was based on the rationale of inducing rapid and profound suppression of the central nervous system, thereby ensuring a humane and peaceful passing for the animals.

Preparation of Mouse Abdominal Skin: Healthy mice were selected, and their abdominal fur was shaved. After euthanizing the mice, the abdominal skin was swiftly excised, eliminating subcutaneous connective tissue and fat. The skin was thoroughly rinsed with physiological saline, yielding mouse abdominal skin. Subsequently, the skin was wrapped in cling film and aluminum foil, then stored at −20 °C for later use [19].

Diffusion Cell Conditions: A Franz diffusion cell was employed with a transdermal area of 0.64 cm², an acceptor chamber volume of 5.0 mL, maintained at a constant temperature of 37 °C in a water bath. The electromagnetic stirring speed was set at 150 rpm, and the receiving fluid used was pH 7.4 phosphate-buffered saline.

Transdermal Permeation Experiment [20]: The thawed ex vivo mouse skin was rinsed with physiological saline, cut into suitable sizes, and fixed with the stratum corneum facing the donor chamber in the Franz diffusion cell. Precisely measured quantities (1 g) of ethosomes gel, ordinary gel, and cream were uniformly applied to the stratum corneum of the skin, with three replicates for each group. Samples were collected at 0, 0.5, 1, 2, 4, 6, 8, 10, 12, and 24 hours (h), taking 1 mL each time, and immediately replenished with an equal amount of receiving fluid at the same temperature. The samples were filtered through a 0.22 μm microporous membrane, and the filtrate was analyzed by injecting it under the conditions specified in section “Chromatographic and mass spectrometric conditions”. The peak area was recorded, and the Huperzine A mass concentration was determined by fitting the values into the standard curve.

The percutaneous permeation curve was obtained by plotting the cumulative amount of Huperzine A permeated per unit area of skin against time.

#### Percutaneous residual amount

The skin was washed with normal saline after the percutaneous test, and then dried and cut into small pieces. The skin pieces were placed in a centrifuge tube with 2 mL of methanol and subjected to sonication and vortexing. The filtered solution was analyzed using the conditions described in section “Chromatographic and mass spectrometric conditions” to determine the concentration. The amount of drug retained in the skin per unit area (Qs) was calculated [21].

#### Data processing and analysis

The cumulative amount of drug per unit area Q_n_ (μg · cm^−2^) was calculated using the following formula:

$${\text{Q}}n = (V \times Cn + \sum\limits_{i = 1}^{n - 1} {Ci \times Vi} )/A$$

**Fig. 3:**
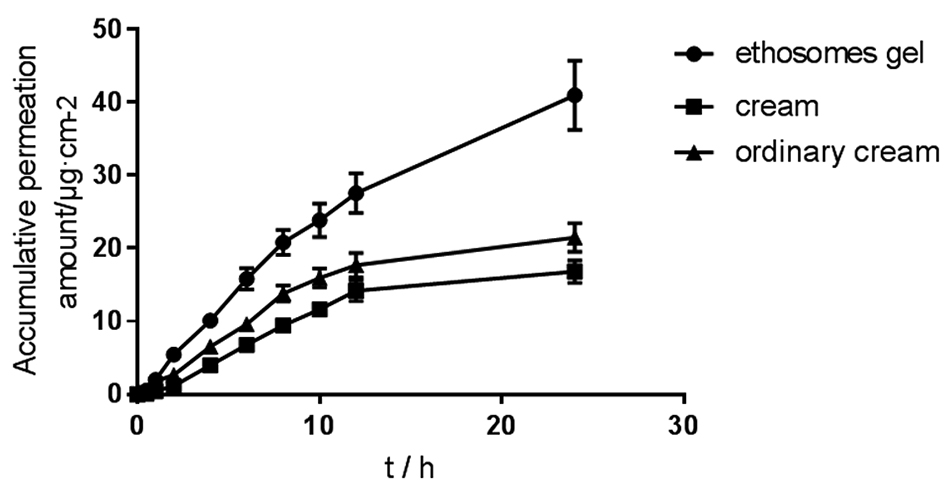
Cumulative penetration curves of Huperzine A ethosomes gel, ordinary gel and cream (${\bar{\text{x}}} \pm {\text{s}}$, n = 3)

In the equation,Q_n_ represents the cumulative permeation amount per unit area at the nth time point, A is the effective permeation area (0.64 cm^2^), C_n_ and C_i_ represent the drug concentrations (μg · cm^−2^) measured at the nth and ith sampling points, respectively, V and Vi represent the volume of the receiving solution (5 mL) and the sampling volume (1 mL) at each time point. The Q_n_–t curves were plotted with Q_n_ as the vertical axis and time t as the horizontal axis. The slope of the curves represents the steady-state permeation rate Js (μg · cm^−2^ · h^−1^), as shown in Fig. 3, Tables 3 and 4.

**Table 3:** Cumulative penetration of Huperzine A (${\bar{\text{x}}} \pm {\text{s}}$, n = 3)

t/h	Huperzine A/μg · cm^−2^		
	Ethosomes gel	ordinary gel	cream
0.5	0.504 ± 0.012	0.056 ± 0.006	0.182 ± 0.014
1	1.982 ± 0.188	1.820 ± 0.198	0.448 ± 0.046
2	5.443 ± 0.507	2.646 ± 0.191	1.134 ± 0.227
4	10.114 ± 0.266	6.538 ± 0.770	3.990 ± 0.352
6	15.792 ± 1.491	9.590 ± 0.899	6.790 ± 0.468
8	20.832 ± 1.760	13.818 ± 1.112	9.422 ± 0.837
10	23.856 ± 2.304	15.946 ± 1.348	11.648 ± 0.972
12	27.552 ± 2.789	17.682 ± 1.690	14.182 ± 1.428
24	40.992 ± 4.826	21.490 ± 1.988	16.800 ± 1.571

T-test (Q_24_): Ethosomes gel vs. Ordinary gel, *p* = 0.0029 (*p* < 0.01)

T-test (Q_24_): Ethosomes gel vs. cream, *p* = 0.0012 (*p* < 0.01)

**Table 4:** Permeation kinetic parameters of Huperzine A in ethosomes gel, ordinary gel, and cream (n = 3)

Formulation	Q-t equation	R^2^	Q_24_/μg · cm^−2^	J/μg · cm^−2^ · h^−1^	Qs/μg · cm^−2^
Ethosomes gel	Q = 1.802 t + 2.541	0.9474	40.992 ± 4.826	1.802	10.159 ± 1.213
Ordinary gel	Q = 0.9968 t + 2.230	0.8584	21.490 ± 1.988	0.9968	4.856 ± 0.356
Cream	Q = 0.8005 t + 1.056	0.8787	16.800 ± 1.571	0.8005	3.089 ± 0.153

To compare the zero-order kinetic characteristics of drug skin permeation among the ethosomes gel, ordinary gel, and cream formulations, linear regression was performed on the Qn–t curves within the 0–12 h and 24 h intervals. The linear fit correlation coefficients r_24_^2^ and r_12_^2^ were obtained and are presented in Table 5. The r_24_^2^ of the ethosomes gel formulation significantly exceeded that of the ordinary gel and cream formulations (*P* < 0.01), while the r_12_^2^ exhibited no significant difference compared to the ordinary gel and cream formulations (*P* > 0.05). These results suggest that the skin permeation of the ethosomes gel formulation adheres more closely to zero-order kinetics and demonstrates a more stable drug permeation rate through the skin, in comparison to the other two formulations.

**Table 5:** In vitro drug permeation parameters of ethosomes gel, ordinary gel, and cream after 12 and 24 h (${\bar{\text{x}}} \pm {\text{s}}$, n = 3)

formulation	Q_24_-t equation	r_24_^2^	Q_12_-t equation	r_12_^2^
Ethosomes gel	Q = 1.802 t + 2.541	0.9474	Q = 2.397 t + 0.2026	0.9920
Ordinary gel	Q = 0.9968 t + 2.230	0.8584	Q = 1.573 t − 0.03893	0.9899
Cream	Q = 0.8005 t + 1.056	0.8787	Q = 1.229 t − 0.6307	0.9954

T-test (r_24_^2^): Ethosomes gel vs. ordinary gel, *p* = 0.0011 (*p* < 0.01)

T-test (r_24_^2^): Ethosomes gel vs. cream, *p* = 0.0028 (*p* < 0.01)

## Discussion

Ethosomes, a new type of liposomes, contain 20–50% small molecule alcohols. The ethanol content in ethosomes alters the arrangement of intercellular lipids in the stratum corneum, increasing its fluidity. Ethosomes possess flexibility and deformability, enabling them to penetrate the disordered stratum corneum and reach deeper skin layers. They can aid in forming drug depots in the skin, leading to a relatively constant release rate [22–24]. Additionally, ethosomes can be incorporated into gels, creams, patches, and other formulations, showing promising potential for transdermal drug delivery [25].

Currently, ethosomes gel has limited application in traditional Chinese medicine, and no marketed products exist. The investigation of the basic properties of Huperzine A ethosomes gel showed that it meets the requirements outlined in the Chinese Pharmacopoeia, demonstrating stable quality and easy application. Huperzine A exhibits rapid metabolism rates when administered orally or through injections, with respective half-lives of 4 and 2.5 h [1]. To ensure therapeutic efficacy, frequent dosing is necessary. However, patients often have difficulties adhering to complex dosing regimens, resulting in fluctuating drug concentrations and increased risk of adverse reactions. Developing a topical formulation for transdermal drug delivery can effectively address this issue, enhancing patient compliance and reducing the risk of peripheral cholinergic system toxicity associated with Huperzine A. The successful development and commercialization of this drug holds great promise.

The in vitro skin permeation study revealed the enhanced permeation characteristics of Huperzine A ethosomes gel. (1) The Q_24_ of ethosomes gel was significantly higher at (40.992 ± 4.826) μg · cm^−2^ compared to ordinary gel (21.490 ± 1.988) μg · cm^−2^ and cream (16.800 ± 1.571) μg · cm^−2^ (*P* < 0.01), indicating a significant increase in transdermal permeation of Huperzine A. This suggests that with an appropriate application area on the skin, the ethosomes gel can achieve the desired daily transdermal dose. The clinical therapeutic dose is 200 μg per day. (2) The r_24_^2^ of ethosomes gel was significantly higher than that of ordinary gel and cream (*P* < 0.01), indicating that compared with ordinary gel and cream, the skin penetration of ethosomes gel was more in line with zero order kinetics, and the transdermal penetration rate of drugs was more stable. (3) The skin retention Qs of ethosomes gel was (10.159 ± 1.213) μg · cm^−2^, which was higher than that of ordinar gel (4.856 ± 0.356) μg · cm^−2^ and cream (3.089 ± 0.153) μg · cm^−2^. indicating its ability to increase drug retention in the skin and achieve sustained release. The Huperzine A ethosomes gel demonstrated a promising percutaneous effect and holds excellent application prospects.

## Data Availability

All data generated or analysed during this study are included in this published article.
